# Modeling photocatalytic degradation of diazinon from aqueous solutions and effluent toxicity risk assessment using *Escherichia coli* LMG 15862

**DOI:** 10.1186/s13568-018-0589-0

**Published:** 2018-04-18

**Authors:** Ali Toolabi, Mohammad Malakootian, Mohammad Taghi Ghaneian, Ali Esrafili, Mohammad Hassan Ehrampoush, Mohsen AskarShahi, Maesome Tabatabaei

**Affiliations:** 1 Environmental Science and Technology Research Center, Department of Environmental Health Engineering, Shahid Sadoughi University of Medical Sciences, Yazd, Iran; 2 Environmental Health Engineering Research Center, Kerman University of Medical Sciences, Kerman, Iran; 3 Department of Environmental Health Engineering, School of Public Health, Kerman University of Medical Sciences, Kerman, Iran; 4 Department of Environmental Health Engineering, School of Public Health, Iran University of Medical Sciences, Tehran, Iran; 5 Department of Biostatistics and Epidemiology, Shahid Sadoughi University of Medical Science, Yazd, Iran; 6 Department of chemistry, Islamic Azad University, Yazd, Iran

**Keywords:** Modeling, Diazinon, Dehydrogenase enzyme, Effluent bioassay

## Abstract

In this study, modeling and degradation of diazinon from contaminated water by advanced oxidation process together with a new test for effluent bioassay using *E. coli* were investigated. The experiments were designed based on response surface methodology. Nanoparticles (NPs) were synthesized using the sol–gel method. The shape characteristics and specifications of elements in the nanoparticles were characterized using scanning electron microscope and energy dispersive X-ray, respectively. Diazinon was measured using high performance liquid chromatography device and by-products due to its decomposition were identified by gas chromatography-mass (GC–MS). In the present study, effluent bioassay tests were conducted by defining the rate of dehydrogenase enzyme reducing alamar blue method. According to statistical analyses (R^2^ = 0.986), the optimized values for pH, dose of NPs, and contact time were found to be 6.75, 775 mg/L, and 65 min, respectively. At these conditions, 96.06% of the diazinon was removed. Four main by-products, diazoxon, 7-methyl-3-octyne, 2-isopropyl-6-methyl-4pyrimidinol and diethyl phosphonate were detected. According to the alamar blue reducing (ABR) test, 50% effective concentration, no observed effect concentration, and 100% effective concentration (EC_100_) for the mortality rate of *E. coli* were obtained as 2.275, 0.839, and 4.430 mg/L, respectively. Based on the results obtained, it was found that mentioned process was high efficiency in removing diazinon, and also a significant relationship between toxicity assessment tests were obtained (P < 0.05).

## Introduction

Organophosphate pesticides (OPs) are among the largest and most diverse types of available pesticides. Considering that they affect a wide range of insects and rodents, these pesticides are used by farmers more than other types. But due to the lack of familiarity with the damaging effects of these toxins or proper principles of combating pests, most consumers do this job either incompletely or indiscriminately (Fadaei et al. 2012; Li et al. 2015; Maddah and Hasanzadeh 2017). Therefore, intentional or unintentional human exposure is as a result of the use of pesticides or their residuals in environments including air, water, soil, and plants. Considering global statistics, the largest portion of mortality from pesticides is related to these toxins. Diazinon is an organophosphate pesticides with pKa = 2.6 and medium risk (Kalantary et al. 2014). The major effects of diazinon on vertebrate life are inhibition of acetyl cholinesterase, resulting in aggregation of acetylcholine in acetylcholine receiver and hyper excitation of nerves and muscles. So far, various technologies have widely been applied for removal of diazinon in aqueous solution such as adsorption, electrocoagulation and biodegradation (Amooey et al. 2014; Ehrampoush et al. 2017).

Since conventional water and wastewater treatment processes are not very effective on the degradation of diazinon (Amooey et al. 2014; Kalantary et al. 2014; Ehrampoush et al. 2017), Recently, advanced oxidation process such as UV/H_2_O_2_, H_2_O_2_/Fe^2+^, NP_S_/UV and etc., due to high efficiency, low cost, and non-toxicity have been considered. Li et al. used UV and UV/H_2_O_2_ process for the removal of diazinon from water resources (Li et al. 2015). Also, Kalantary et al. successfully used TiO_2_/UV process for the degradation of diazinon (Kalantary et al. 2014). TiO_2_ nanoparticles along with UV, have been considered as an effective method for water treatment (Amooey et al. 2014; Li et al. 2015; Ribeiro et al. 2015; Ehrampoush et al. 2017; Maddah and Hasanzadeh 2017). The energy of light from UV rays in contact with titanium atoms, stimulates its surface electrons and moves them from the valence layer to the conductive layer, The result of this energy change will be the formation of a halo at the surface of the titanium atom and the formation of free electrons  (OH^•^). These active radicals cause oxidation of organic matter in the solution and convert it to water and carbon dioxide. One of the disadvantages of titanium nanocatalysts is the existence of an inter-structural hole in this composition. This means that a less energy band of ultraviolet radiation will remain on the surface of the catalyst (Mohammadi and Sabbaghi 2014; Tian et al. 2014; Toolabi et al. 2017; Wang and Shih 2016). Accordingly, in the current study, to enhance the optimal response of titanium dioxide, silica dioxide was introduced to the reaction. Performing the effluent toxicity risk assessment after water treatment processes is essential for environmental, drinking water and public health. Previously, to determine the effluents toxicity, some methods such as tetrazolium salt, crystal violet, and colony forming unit were used. But often they were expensive, long-term, and unreliable (Pettit et al. 2005; Satyanarayan et al. 2016). Recently, alamar blue (AB) due to its high sensitivity and non-toxicity has been widely used in studies on bioassay in a biological range such as bacteria, piscine cells and planktonic assays (Rampersad 2012; Khalifa et al. 2013; Teh et al. 2017).

Because the oxidation–reduction potential (ORP) of alamar blue is more than the enzyme dehydrogenase, it was reduced by the dehydrogenase enzyme. But in the presence of living bacteria, alamar blue is converted to resorufin and the color of the solution changes from blue to pink (Nasiry et al. 2007; Rampersad 2012; Gregoraszczuk et al. 2015; Balouiri et al. 2016; Tyc et al. 2016; Zare et al. 2016; Teh et al. 2017; Toolabi et al. 2017). To achieve the best and most effective method of removal and risk assessment of diazinon in aqueous solution, more studies need to be done in this regard. Therefore, in this study, application of Fe_3_O_4_/SiO_2_/TiO_2_/H_*2*_O_2_/UV-C process for the degradation of diazinon and novel test for the effluent toxicity risk assessment using *Escherichia coli* were conducted.

## Materials and methods

### Chemicals and media

Analytical diazinon pesticide with a purity of 98.5%, Acetic acid 99.9%, ethanol 99.9%, chloride iron (II), chloride iron (III), tetra ethyl ortho silicate 95%, tetra-n-butyl lorthotitanate, ammonium solution, alamar blue powder, agar muller hinton, broth nutrient, dimethyl sulfur oxide (DMSO), n-amyl alcohol, HCl-phthalate buffer, glucose, sodium acetate, sodium bicarbonate, Sulfuric acid 98%, Sodium Hydroxide 98%, potassium phosphate monobasic and Dipotassium phosphate were purchased from Sigma Aldrich Co. The properties of diazinon and alamar blue are shown in Table 1.

**Table 1:**
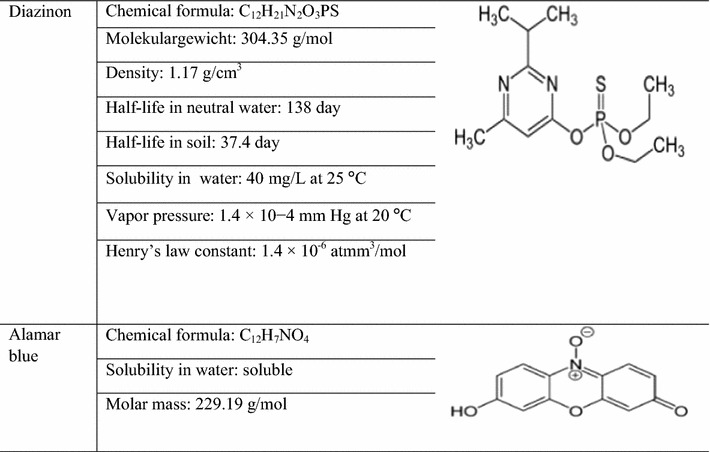
Properties of diazinon and alamar blue

### Microorganism

A standard strain of *Escherichia coli* LMG 15862 bacteria was purchased from Tehran Razi Institute and immediately was stored at a temperature of 8 °C.

### Synthesis of nanoparticles

There are various methods for synthesizing and doping TiO_2_/Fe_3_O_4_/SiO_2_ nanoparticles. These routes include sol–gel process, co-precipitation, hydrothermal method, pyrolysis spray, sono-chemical synthesis, and wet immersion method (Tian et al. 2014; Gupta et al. 2015).

### Fe_3_O_4_ nanoparticles

The synthesis of Fe_3_O_4_ nanoparticles was done according to co-precipitation method. Briefly, 23.36 g of chloride iron (III) and 8.62 g of chloride iron (II) were dissolved in 250 cc of deionized water for 50 min and mixed at 87 °C inside a reactor (Cylindrical and quartz glass with a diameter of 35 cm and length of 45 cm). Thereafter, the resulting solution was slowly injected into 3.6 L of deionized water. Next, action bubbling of nitrogen gas was conducted for 24 h  at 75 °C. After these three stages of washing with water and ethanol, Fe_3_O_4_ nanoparticles were formed (Shunxing et al. 2016; Maddah and Hasanzadeh 2017; Toolabi et al. 2017).

### Fe_3_O_4_/SiO_2_/TiO_2_ nanoparticles

The synthesis of nanoparticles was done using the sol–gel method. The nanoparticles obtained in the previous step were dissolved in 250 cc deionized water containing tetraethyl orthosilicate, in the next step, ultrasonic (Hielscher model, Sonication of liquids 0.5–4.0 L/min) was used to better separate the nanoparticles. Thereafter, for transparency of nanoparticles and crystal formation, 30 mL of acetic acid was added to the reactor containing nanoparticles of iron/silica and mixed at 200 rpm. Next, the combination of acetic acid, ethanol and tetra-n-butyl lorthotitanate was prepared. The mixture obtained was added to the heater reactor and mixed at 500 rpm. After three stages of washing with deionized water and ethanol, Fe_3_O_4_/SiO_2_/TiO_2_ was formed (Shunxing et al. 2016; Toolabi et al. 2017; Wang et al. 2017). The surface and shape characteristics of the nano composite and quantitative analysis of the elements were characterized using a scanning electron microscope and energy dispersive X-ray, respectively.

### Modeling and statistical analysis

In this work, to model and design the experiments, response surface methodology (RSM) was used. This model is a collection of statistical and mathematical techniques that are useful for analyzing the effects of several independent variables on a response. RSM is an effective statistical technique for optimizing the number of experiments. Also, it specifies the interconnected amount of variables and the most optimal variable is presented in order of preference. RSM contains various models such as the Behnken design, central composite design (CCD), factorials method, box-d-optimal design etc. (Martino et al. 2015; Sarrai et al. 2016; Dehghani et al. 2017; Nama et al. 2018). In the present study, according to the CCD model, the number of experiments was designed for variables such as diazinon concentration (1–40 mg/L), contact time (10–120 min), pH (3–12), and dose of nanoparticles (100–1000 mg/L), Table 2. Expert Design Ver 7 was used for the data analysis.

**Table 2:** The levels of variables central composite statistical experiment design

Factor	Variables	Low actual	High actual	Mean	Std. Dev.
A	pH	2.012	7.500	9.75	5.25
B	Contact time (min)	24.597	65.000	92.50	37.50
C	Concentration of diazinon(mg/L)	8.721	20.500	30.25	10.75
D	Dose of NPs (mg/L)	201.246	550.000	775.00	325.00

### Analytical procedures

Experiments were conducted inside a glass reactor (11 × 11 × 25 cm) with a reflective wall. This reactor was equipped with a UV lamp (λ = 254 nm, P = 125 W, L = 10 cm) surrounded by a quartz tube, cooling system, an air blower pump with a flow rate of 3 L per minute to remove gases from the reactor and also to prevent possible precipitation of nanoparticles to the bottom of the reactor, pH meter and multipara meter device. Also a radiometer device (model Hanger ECL-X) was used to measure the intensity of UV radiation. During the experiment process, sampling was done based on the CCD. In order to increase the production of radical hydroxyl ion in a solution, H_2_O_2_ compound was used at a concentration of 50 mg/L (Shemer and Linden 2006). All samples filtered by using a syringe equipped with a 0.2 micron filter. The concentration of diazinon was measured using High Performance Liquid Chromatography (HPLC), the following specifications were used; wavelength was 260 nm, C18 column, length and diameter of the column were 4.6 × 250 mm and the volume of injection sample = 20 µL. The removal efficiency of diazinon was obtained using the following Eq. 1.

1 $${\text{Removal }}\left( \% \right) = \left( { 1 { }{-}{\text{ C}}_{\text{t}} /{\text{C}}_{\text{o}} } \right)\;\times\;100$$

where C_o_ is the initial concentrations of diazinon (mg/L) and C_t_ is the residual of diazinon (mg/L) after the specified time.

By-products resulting from the degradation of diazinon were detected using gas chromatography-mass (GC–MS) model Agilent Technologies 19091S-433 with a HP-5MS column (length 25 m, thickness 0.25 mm, diameter 0.25 mm) (Ehrampoush et al. 2017; Toolabi et al. 2017).

### Chemical oxygen demand (COD)

Based on the standard methods in the purification of water sources, the rate of mineralization of diazinon was determined by measuring the COD. Accordingly, COD removal was determined using Eq. 2.

2 $$\% {\text{COD Removal = }}\left( {\frac{{{\text{COD}}_{\text{in}} - {\text{COD}}_{\text{r}} }}{{{\text{COD}}_{\text{in}} }}} \right)$$

where COD_in_ is initial COD (mg/L) and COD_r_ is the COD (mg/L) residual concentration according to CCD parameters.

### Toxicity assessment based on ABR methods

The rate of alamar blue dye reduction was determined by the activity of enzyme dehydrogenase; first broth nutrient culture medium was enriched with KH_2_PO_4_ (3.28 g/L), K_2_HPO_4_ (5.28 g/L), sodium acetate (0.4 g/L) and glucose (0.4 g/L). Next, 2 mL of *E. coli* suspension and 2 mL of alamar blue solution with concentration of 200 mg/L were added to the broth nutrient medium. Then, 1 mL of the diazinon was added with specific concentrations. Next, it was incubated at 30 °C under darkness condition. Following 60 min of contact time, 2 mL of HCl-phthalate 0.05 M buffer and 20 mL of n-amyl alcohol solution were added to each test tube. Afterwards these materials were stirred slowly, The rate of alamar blue reduction was determined through the extent of absorption at the wavelength of 620 nm using UV/ViS spectrophotometer device (Braic 2100) (Toolabi et al. 2017; Zare et al. 2016).The percentage of alamar blue reduction was obtained using Eq. 3.

3 $${\text{Reduce activity of dehydrogenase enzyme in alamar blue conversion }}\left( \% \right) = \left( {{\text{A }} - {\text{ B}}} \right)/{\text{A}} \times 100$$

where A is the rate of activity of dehydrogenase enzyme in the control sample and B is the rate of activity of dehydrogenase enzyme in the main sample.

### Toxicity assessment based on CFU methods

To investigate the validity of ABR test and effluent bioassay, CFU test was conducted. Accordingly, first, a suspension of *E. coli* LMG bacteria was prepared. Suspension turbidity was detected using spectrophotometer device. Based on 0.5 McFarland, optical density (OD) 0.6 was generated. By measuring the turbidity in the suspension, the density of the bacterial cells was obtained in the range of 2–3 × 10^8^ cells/mL. To determine the mortality rate of *E. coli* bacteria, 100 µL of bacterial suspension was injected on a plate containing the Mueller–Hinton medium and diazinon (Nasiry et al. 2007; Gregoraszczuk et al. 2015; Balouiri et al. 2016; Tyc et al. 2016; Toolabi et al. 2017). After 24 h of incubation, the growth inhibition percentage was determined by Eq. 4.

4 $${\text{Growth inhibition percentage}} = {\text{A}} - {\text{B}}/{\text{A}} \times 100$$

where A is the number of colonies of the control sample and B is the number of colonies of the inoculated sample. Finally, for both tests (ABR and CFU), the results were reported as follows: The amount of toxin required for decreasing the growth less than 1% of the bacteria initial population was reported as no observed effect concentration (NOEC), the amount of toxin required for decreasing 50 and 100% of the bacterial growth was reported as effective concentration (EC_50_) and effective concentration (EC_100_), respectively.

### Sampling from natural source

After determining the optimal parameters for removal of diazinon by advanced oxidation process, sampling of water from the Seymareh River was carried out for 6 consecutive months. Sampling was carried out once a week and the volume of each sample 2 L was selected. After the samples were transferred to the laboratory, their physical and chemical characteristics were determined. Samples were introduced into the photocatalyst reactor. And removal efficiency of diazinon were obtained under optimum conditions. Then, Alamar Blue reduction and colony count unite tests were used to determine the toxicity of the effluent.

## Results

### Scanning electron microscopy

**Fig. 1:**
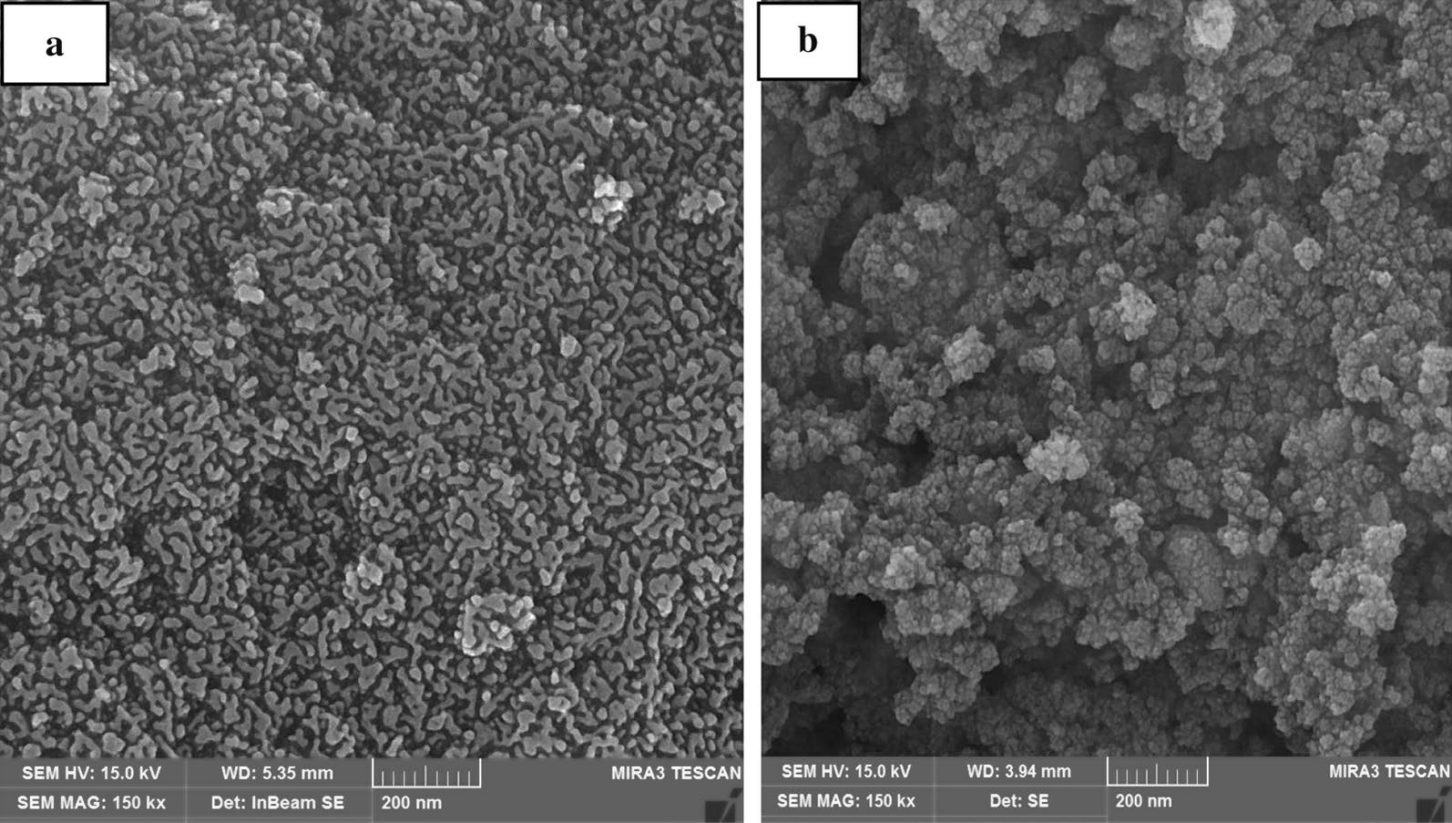
The results of SEM images of **a** Fe_3_O_4_ nanoparticles and **b** Fe_3_O_4_/SiO_2_/TiO_2_ nanoparticles

Information on surface morphology and particle size distribution of Fe_3_O_4_ and Fe_3_O_4_/SiO_2_/TiO_2_ were characterized using Scanning electron microscopy, Fig. 1. Accordingly, the high transparency of nanoparticles production was achieved with energy of 15 kV and their accumulation properties were not observed. Also, according to the analysis of the size of the nanoparticles, the typical size of nanoparticles was determined to be 200 nm.

### Energy dispersive X-ray spectroscopy

**Fig. 2:**
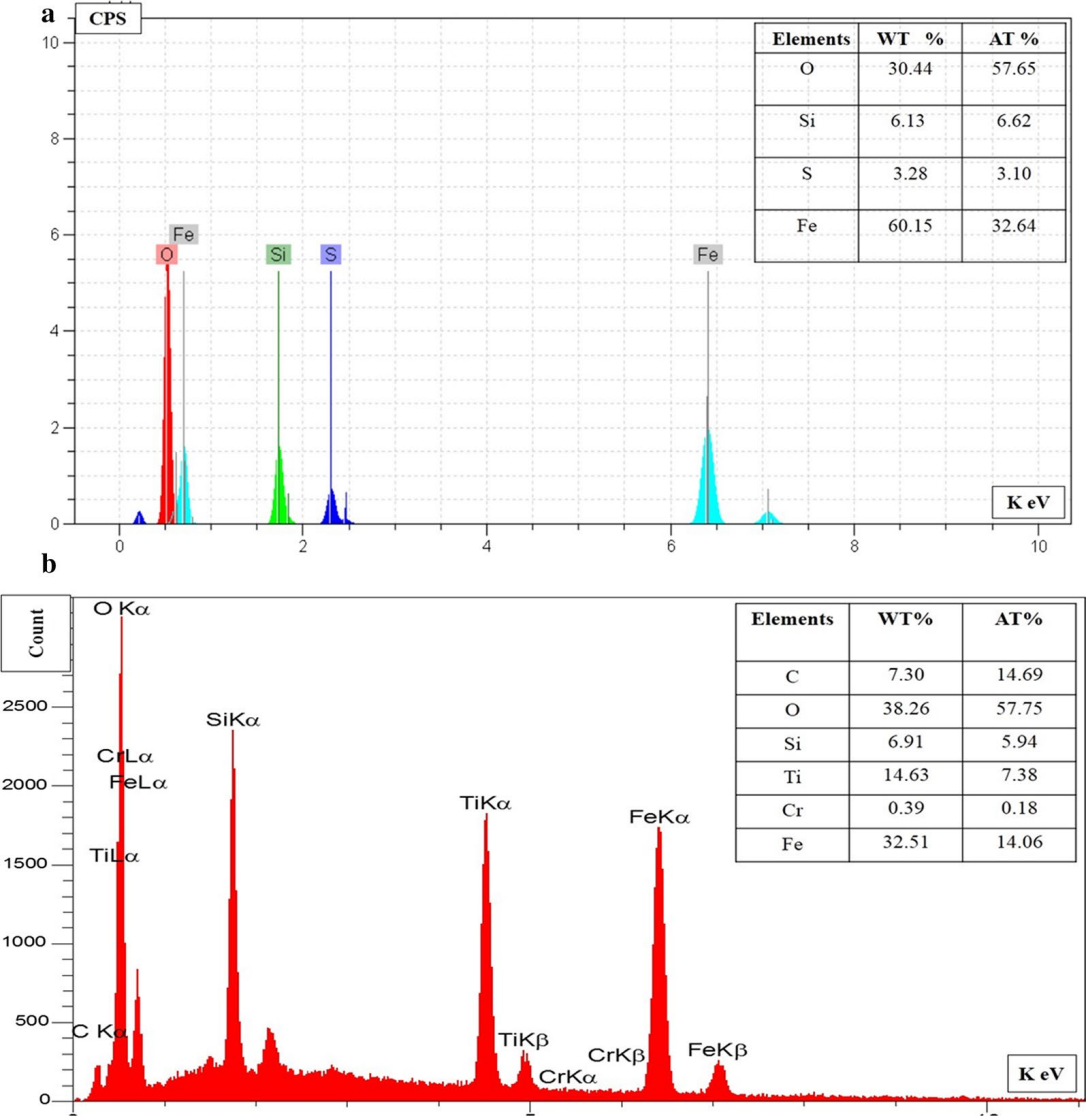
EDX spectrum of **a** Fe_3_O_4_/SiO_2_ and **b** Fe_3_O_4_/SiO_2_/TiO_2_ nanoparticles

According to Fig. 2, elemental composition analysis using EDX was presented at 0.2 to 8 keV. In Fe_3_O_4_/SiO_2_ composite, O, Fe, Si, and S elements were diagnosed Fig. 2a. The weakest and strongest signals were related to S and Fe elements, respectively. It was also shown in Fig. 2b that Fe_3_O_4_/SiO_2_/TiO_2_ nanoparticles contain O, C, Fe, Si, Ti, S, and Cr elements. The weakest and strongest signals were related to Cr and O, respectively.

### Statistical analysis and modeling

**Fig. 3:**
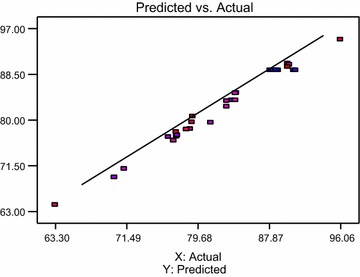
The relationship between real values and predicted values

According to the central composite design, the number of 30 runs was designed and the efficiency removal of diazinon belonging to each run was determined, Table 3. The optimum run was related to run 27; in this case, the removal efficiency of diazinon was reported to be 96.06%. Also, the predicted value of each run was determined. Accordingly, a direct relationship between real values and predicted values was reported Fig. 3 (R^2^ = 0.943). Further details are shown in Table 3.

**Table 3:** Results of the experimental runs based on the central composite design

Run	A:PH	B:Contact time (min)	C:Concentration of diazinon (mg/L)	D:Dosage of nanoparticles (mg/L)	Removal efficiency (%)	Predicted value
1	7.500	65.00	20.50	550.0	88.90	89.26
2	7.500	65.00	20.50	550.0	88.68	89.26
3	7.500	65.00	20.50	550.0	87.99	89.26
4	7.500	65.00	20.50	550.0	88.39	89.26
5	7.500	65.00	20.50	550.0	90.91	89.26
6	7.500	65.00	20.50	550.0	90.70	89.26
7	5.250	37.50	10.75	325.0	83.60	83.67
8	9.750	37.50	10.75	325.0	77.24	76.92
9	5.250	92.50	10.75	325.0	84.10	85.02
10	9.750	92.50	10.75	325.0	77.33	77.19
11	5.250	37.50	30.25	325.0	76.30	76.86
12	9.750	37.50	30.25	325.0	70.10	69.36
13	5.250	92.50	30.25	325.0	81.17	79.52
14	9.750	92.50	30.25	325.0	71.23	70.93
15	5.250	37.50	10.75	775.0	90.00	90.52
16	9.750	37.50	10.75	775.0	84.00	84.99
17	5.250	92.50	10.75	775.0	90.20	90.28
18	9.750	92.50	10.75	775.0	84.00	83.66
19	5.250	37.50	30.25	775.0	83.00	82.47
20	9.750	37.50	30.25	775.0	76.90	76.20
21	5.250	92.50	30.25	775.0	83.00	83.54
22	9.750	92.50	30.25	775.0	76.91	76.18
23	3.000	65.00	20.50	550.0	78.81	78.33
24	12.00	65.00	20.50	550.0	63.30	64.22
25	7.500	10.00	20.50	550.0	78.39	78.25
26	7.500	65.00	20.50	550.0	79.00	79.59
27	7.500	65.00	1.000	550.0	96.06	94.95
28	7.500	65.00	40.00	550.0	79.10	80.66
29	7.500	65.00	20.50	100.0	77.20	77.78
30	7.500	65.00	20.50	1000	90.00	89.87

In this study, the regression results of quadratic, linear, 2FI, and cubic models for the removal efficiency of diazinon is shown in Table 4. Accordingly, for R^2^ = 0.9865, the quadratic model was more credible than other models. The final equation to describe the actual factors according to the quadratic model is shown in Eq. 5.

**Table 4:** The results of Statistics Model

Source	Std. Dev	R^2^	Adjusted R^2^	Predicted R^2^	PRESS
Linear	5.313	0.5397	0.4660	0.3788	952.3
2FI	6.056	0.5455	0.3063	0.2559	1141
Quadratic	1.174	0.9865	0.9739	0.9438	86.20
Cubic	1.135	0.9941	0.9756	0.8630	210.0

5 $$\begin{aligned} {\text{Removal efficiency of diazinon}}\% & = 8 9. 2 6- 3. 5 2 8\times {\text{A}} - 0. 3 3 4 2\times {\text{B}} - 3. 5 7 4 \\ & \quad \times\; {\text{C}} - 3.0 2 3\times {\text{D}} - 0. 2 7 1 3 \times {\text{AB}} - 0. 1 8 7 5\times {\text{AC}} - 0. 30 50 \times {\text{AD}} - 0. 3 2 6 2\times {\text{BC}} - 0. 3 9 8 8 \\ & \quad \times \; {\text{BD}} - 0. 30 7 5\times {\text{CD }} - 4. 4 9 6\times {\text{A}}^{ 2} - 2. 5 8 6\times {\text{B}}^{ 2} - 0. 3 6 4 6 { } \times {\text{ C}}^{ 2} - 1. 3 60 \times {\text{D}}^{ 2} \\ \end{aligned}.$$

Based on Eq. 5, the maximum removal percentage of diazinon 96.06 was obtained. Impact coefficient for variables such as pH, contact time, diazinon concentration, and dose of NPs was obtained 3.528, 0.3342, 3.574 and 3.023, respectively. As shown in Eq. 5, the main parameter is related to the pH variable. Also, the minimum and maximum interaction amount variables in relation to the coefficient of AC and BD Coded Factors were obtained as 0.1875 and 0.3988, respectively. In this study, the F-value, P value and degree of freedom (DF) parameters were conducted for the analysis of variance. According to the results shown in Table 5, the F-value, P-value and DF were obtained as 78.32, < 0.0001, and 14, respectively.

**Table 5:** ANOVA of Response Surface Quadratic Model

Source	Sum of squares	Df	Mean square	F value	P value Prob > F
Model	1512	14	108	78.32	< 0.0001 significant
A	298.8	1	298.8	216.6	< 0.0001
B	2.680	1	2.680	1.943	0.1836
C	306.6	1	306.2	222.3	< 0.0001
D	219.3	1	219.3	159.0	< 0.0001
AB	1.177	1	1.177	0.853	0.3702
AC	0.562	1	0.562	0.407	0.5327
AD	1.488	1	1.480	1.079	0.3154
BC	1.703	1	1.703	1.235	0.2840
BD	2.544	1	2.540	1.844	0.1945
CD	1.513	1	1.513	1.097	0.3154
A^2^	554.4	1	554.4	401.9	< 0.0001
B^2^	183.4	1	183.4	133.0	< 0.0001
C^2^	3.645	1	3.646	2.643	0.1248
D^2^	50.70	1	50.70	36.76	< 0.0001
Residual	20.69	15	1.379	–	–
Lack of fit	13.06	10	1.306	0.855	0.617 not significant
Pure error	7.63	5	1.526	–	–
Cor total	1533	29	–	–	–

*Df* Degree of freedom

### Effect of variables on the removal efficiency

**Fig. 4:**
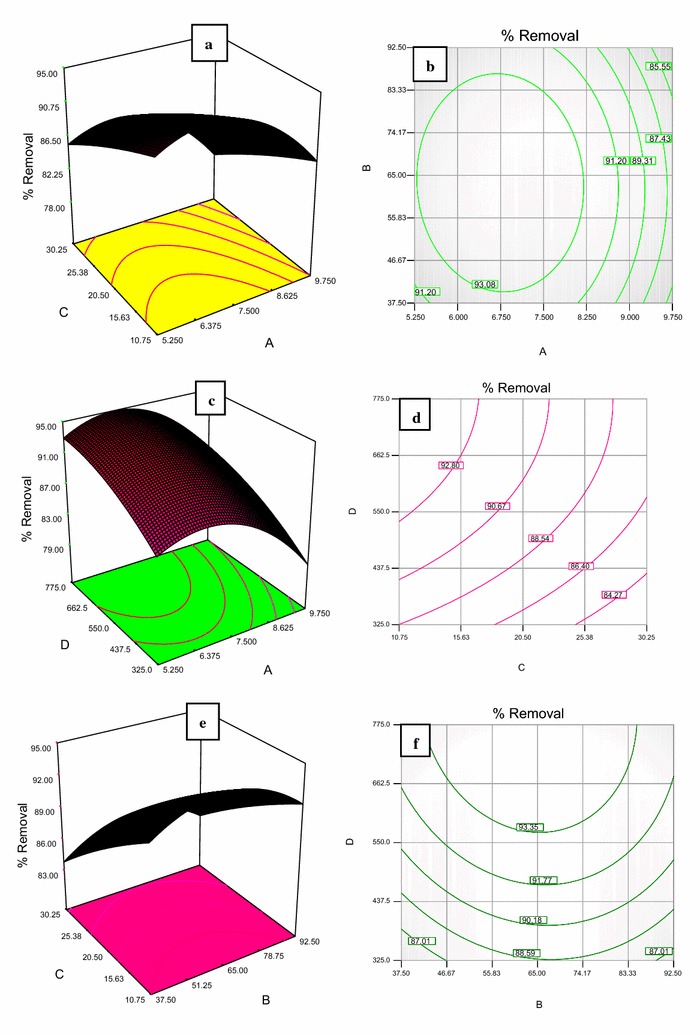
Contour model and 3-D response for removal of diazinon with interactions among factors, **a** contact time = 65 min and dose of NPs = 775 mg/L, **b** dose of NPs = 775 mg/L, concentrations of diazinon = 10.75 mg/L, **c** concentration of diazinon = 10.75 mg/L and contact time = 65 min, **d** pH = 6.5 and contact time = 65 min, **e** pH = 6.5 and dose of NPs = 775 mg/L, **f** concentration of diazinon = 10.75 mg/L and pH = 6.5

The results indicated that this process has been highly efficient in the removal of diazinon and COD. As shown in Figs. 3, 4, D response and contour plot models were studied for the removal of diazinon. The effect of the initial concentration of diazinon in the reactor was investigated from 1 to 40 mg/L. As shown in Fig. 4, by increasing the initial concentration of diazinon, the removal efficiency decreased. Accordingly, at pH = 6.75 and contact time = 65 min, by increasing the initial concentration from 10.75 to 30.25 mg/L, removal efficiency of diazinon decreased from 92 to 85%. The optimal pH for diazinon removal was obtained near 7. When pH increased from 6.75 to 9.5, the removal efficiency of diazinon decreased from 90.5 to 82%. Also, in this study, optimal contact time and optimal dose of nanoparticles were obtained in 65 min and 775 mg/L, respectively, Fig. 4.

### Identification of products by GC–MS

**Fig. 5:**
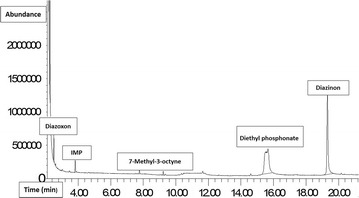
Gas chromatography–mass spectrometry of diazinon

In this study, the analysis of by-products was performed based on the following conditions; pH = 6.75, contact time = 40–80 min, dose of NPs = 775 mg/L and diazinon Concentration = 10.75 mg/L. Speciation and molecular structures of the oxidation by-products were analyzed by GC–MS, Fig. 5. According to the results shown in Table 6, four by-products, including; diazoxon, 7-methyl-3-octyne, 2-isopropyl-6-methyl-4-pyrimidinol (IMP) and diethyl phosphonate were identified during degradation of diazinon. Their retention time (RT) varied from 2.15 to 15.75 min. As such, the minimum and maximum RT were related to diazoxon and diethyl phosphonate compounds, respectively. The characteristics of other compounds are shown in Table 6.

**Table 6:** The characteristics of by-products identification due to diazinon decomposition

Compound name	Molecular formula	Retention time (min)	Molecular weight (g/mol)
Diazoxon	C_12_H_21_N_2_O_4_P	2.15	288.284
2-isopropyl-6-methyl-pyrimidin-4-ol (IMP)	C_8_H_12_N_2_O	3.25	152.197
7-Methyl-3-octyne	C_9_H_16_	7.65	124.223
Diethyl phosphonate	C_4_H_10_O_3_P^+^	15.75	137.095

### Effluent toxicity assessment

In this study, to determine the mortality rate of *E. coli* LMG bacterial, NOEC, effective concentration (EC) parameter was used. Accordingly, the growth inhibitory level Before and after from performing the advanced oxidation process (AOP) was obtained. EC_50_ related to ABR and CFU tests before from performing AOP was obtained as 2.255 and 2.250 mg/L, respectively, Also, NOEC related to ABR and CFU tests was obtained as 0.890 and 0.850 mg/L, respectively, Table 7. Based on the results shown in Table 8, the effluent toxicity assessment from the reactor in different runs for EC_50_ and NOEC parameters related to ABR test after from performing AOP were obtained as 2.275 and 0.839 mg/L, respectively.

**Table 7:** The result of diazinon effect concentration in ABR and CFU tests by using *E. coli*

Parameters	Type of test	Bottom limit	Upper limit	Typical value
EC_5_ (mg/L)	ABR CFU	0.316 0.313	0.329 0.323	0.324 0.319
EC_10_ (mg/L)	ABR CFU	0.519 0.515	0.533 0.529	0.530 0.525
EC_15_ (mg/L)	ABR CFU	0.817 0816	0.829 0.825	0.823 0.820
EC_20_ (mg/L)	ABR CFU	0.959 0.950	1.000 0.987	0.968 0.964
EC_25_ (mg/L)	ABR CFU	1.287 1.272	1.297 1.287	1.293 1.285
EC_30_ (mg/L)	ABR CFU	1.502 1.489	1.513 1.508	1.510 1.500
EC_35_ (mg/L)	ABR CFU	1.612 1.609	1.630 1.621	1.619 1.611
EC_40_ (mg/L)	ABR CFU	1.826 1.812	1.831 1.852	1.826 1.818
EC_45_ (mg/L)	ABR CFU	2.030 2.022	2.041 2.032	2.034 2.027
EC_50_ (mg/L)	ABR CFU	2.230 2.235	2.265 2.255	2.255 2.250
EC_55_ (mg/L)	ABR CFU	2.360 2.339	2.372 2.351	2.363 2.347
EC_60_ (mg/L)	ABR CFU	2.584 2.545	2.591 2.577	2.587 2.571
EC_65_(mg/L)	ABR CFU	2.719 2.761	2.732 2.795	2.724 2.790
EC_70_ (mg/L)	ABR CFU	2.932 2.900	2.961 2.920	2.951 2.911
EC_75_ (mg/L)	ABR CFU	3.100 3.127	3.125 3.149	3.111 3.145
EC_80_ (mg/L)	ABR CFU	3.329 3.351	3.343 3.371	3.335 3.360
EC_85_ (mg/L)	ABR CFU	3.742 3.668	3.659 3.702	3.652 3.689
EC_90_(mg/L)	ABR CFU	3.878 3.890	3.888 3.823	3.881 3.812
EC_95_ (mg/L)	ABR CFU	4.000 3.975	4.116 4.120	4.080 4.020
NOEC (mg/L)	ABR CFU	0.884 0.847	0.893 0.859	0.890 0.850
EC_100_(mg/L)	ABR CFU	4.010 3.980	4.300 4.221	4.128 4.120

**Table 8:** The result of COD Removal, ORP and bioassay test to determination of effluent toxicity in different Runs

Run	COD removal %	ORP (mv)	Residuals of diazinon (mg/L)	Rate of EC, NOEC	
				ABR test	CFU test
1	94.50	333	2.275	EC_50_	EC_49_
2	96.00	330	2.320	EC_55_	EC_56_
3	94.40	325	2.462	EC_62_	EC_59_
4	95.30	322	2.585	EC_65_	EC_56_
5	96.00	339	1.863	EC_40_	EC_37_
6	95.70	337	1.906	EC_42_	EC_38_
7	93.90	338	1.763	EC_41_	EC_35_
8	92.80	327	2.446	EC_58_	EC_53_
9	94.00	341	1.709	EC_37_	EC_33_
10	92.80	331	2.437	EC_55_	EC_50_
11	92.10	–	7.169	EC_100_	EC_100_
12	88.90	–	9.044	EC_100_	EC_100_
13	93.50	–	5.683	EC_100_	EC_100_
14	90.00	–	8.702	EC_100_	EC_100_
15	96.90	349	1.075	EC_22_	EC_19_
16	96.00	340	1.720	EC_39_	EC_35_
17	93.80	350	1.075	EC_20_	EC_18_
18	91.50	340	1.720	EC_40_	EC_37_
19	93.50	–	5.142	EC_100_	EC_100_
20	92.00	–	6.987	EC_100_	EC_100_
21	93.80	–	5.142	EC_100_	EC_100_
22	92.00	–	6.985	EC_100_	EC_100_
23	93.20	–	4.434	EC_100_	EC_100_
24	85.00	–	7.523	EC_100_	EC_100_
25	93.00	–	4.430	EC_100_	EC_100_
26	94.00	301	4.305	EC_98_	EC_96_
27	99.20	360	0.839	NOEC	NOEC
28	94.50	–	8.396	EC_100_	EC_100_
29	92.40	–	4.674	EC_100_	EC_100_
30	96.50	335	2.050	EC_44_	EC_43_

### Analysis of the river water samples

The characteristics of raw water of Seymareh Rive are shown in Table 9, Based on the results of analysis of the River water samples, it was found that the removal efficiency of diazinon by advanced oxidation process is 95%, and it was found that the COD was decreased from 55 to 1.65 mg/L. By analyzing the effluent toxicity Using Alamar blue and colony forming unit tests, it was observed that the number of bacteria are not decreased.

**Table 9:** Characteristics of raw water of Seymareh Rive

Parameters	Value (average)
Temperature (°C)	16.3
Turbidity (NTU)	105
Oxygen dissolve (mg/L)	6.2
BOD (mg/L)	35
COD (mg/L)	55
Concentration of diazinon (mg/L)	1.17

## Discussion

According to the results obtained in Fig. 1, it was found that the syntheses of Fe_3_O_4_/SiO_2_/TiO_2_ nanoparticles were successful. By using SEM techniques, the size of the nanoparticles was confirmed at a range of 200 nm. Also, by comparing the elements and peaks produced by EDX analysis, It was found that sol–gel and co-precipitation methods were acceptable for the synthesis of nanoparticles in this study.

According to the analysis of variance Table 5, values of Prob > F less than 0.0500 show that the model quality is significant. Accordingly, the A, C, D, A_2_, B_2_, and D_2_ parameters are significant. The F value of 78.32 and the Prob > F value of < 0.0001 suggest that the model was statistically approved for removal of diazinon. Also, based on the results obtained from the quadratic model in Table 4, the R^2^ value and Adj R^2^ value were obtained as 0.986 and 0.973, respectively. These results showed that the predicted values obtained from the quadratic model is a fit of the experimental results (Martino et al. 2015; Sarrai et al. 2016; Dehghani et al. 2017).

In order to increase the photo catalytic properties in the process, hydrogen peroxide was added to the reactor. Hydrogen peroxide led to more formation of hydroxyl radicals and resulted in the oxidation of the pesticide compounds (Fadaei et al. 2012; Asaithambi et al. 2017). According to the results obtained in Fig. 4, by increasing the contact time from 37 to 65 min, removal efficiency of diazinon was increased from 85.5 to 91%. This is due to the production of more OH radicals in longer time and also more exposure of active radicals by diazinon; the possibility of the decomposition of a larger percentage of diazinon is provided. Based on the results from the one factor response model, three-dimensional response and contour model, by increasing the concentration of NPs, the removal efficiency of diazinon was increased. Therefore, at a dosage of 320 mg/L of NPs, the removal percentage of diazinon was obtained at 85. Once the dosage of NPs was increased to 775 mg/L, the removal percentage of diazinon reached 92.5. This is because, when the concentration of nanoparticles under the influence of UV radiation is increased in the reactor test, h^+^ and e^−^ ions are produced. Afterwards, these ions react with water and peroxide radicals and also hydroxide ions are produced (Shunxing et al. 2016; Toolabi et al. 2017). Peroxide radicals are mixed with H^+^ ions and hydroxyl radicals (OH^•^) are formed. Due to the high oxidation power of OH radicals, the degradation of the diazinon occurred. In this study, it was found that due to the reflective wall of the reactor, the amount of radiation produced is 1.45 times higher than that of conventional reactors under similar conditions. This causes more electrons to be stimulated from the catalyst surface, and the production of active radicals in the solution increased.

Based on the results of this study, pH was the most effective parameter in removing diazinon. The maximum removal efficiency was obtained when pH was equal to 6.75, this was because more hydrolysis of diazinon occurs in acidic solutions. Also, the production of active hydroxyl radicals is higher in acidic solutions. Therefore, this parameter should be given more attention in future studies (Li et al. 2015; Ehrampoush et al. 2017; Toolabi et al. 2017). In the study of Kalantary et al., optimal parameters such as pH of the nanoparticle and the contact time degradation of diazinon were obtained by using the TiO_2_/UV process at 6, and 550 mg/L, and 60 min, respectively and the maximum removal efficiency of diazinon was obtained as 71% (Kalantary et al. 2014). This difference in the removal of diazinon can be due to the experimental conditions, such as the presence of silica and hydrogen peroxide in the present study.

According to the results of this study, four by-products, diazoxon, 7-methyl-3-octyne, 2-isopropyl-6-methyl-4-pyrimidinol (IMP) and diethyl phosphonate were identified during degradation of diazinon. By increasing the contact time from 40 to 80 min, the major of by-products were disappeared. Also by determining the toxicity of the effluent from the reactor, it was found that the toxicity of these compounds was less than that of diazinon. Similar to this study (Li et al. 2015), IMP was reported as the oxidation product of diazinon during advanced oxidation process, which is less toxic than its parent compound.

Also, In another study conducted by Kalantary et al. (2014), diazoxon and IMP compounds were introduced as by-products due to the diazinon degradation and by assessing their toxicity, it was found that their toxicity is less than that of diazinon. Therefore, according to the results obtained in this study, Fe_3_O_4_/SiO_2_/TiO_2_/H_*2*_O_2_/UV-C process by producing active radicals (OH^•^) can decompose diazinon and its by-products.

According to the results from Table 8, the degree mineralization of diazinon was determined by using COD experiment. Therefore, the minimum and maximum mineralization of diazinon in effluent reactor was obtained as 88.90 and 99.20%, respectively. By increasing the COD removal percentage, the activity of dehydrogenase enzyme was increased. So, a significant statistical relationship with *P*-value < 0.05 between COD decomposition and alamar blue reduction was obtained. Based on the results obtained in this study, there was a direct correlation between ABR and CFU tests (P < 0.05). Accordingly, EC_50_, EC_100_, and no observed effect concentration in the effluent were obtained as 2.255, 4.128, and 0.890 mg/L, respectively Table 7. Also, the effluent from the reactor was evaluated with ABR and CFU tests. According to the results presented in Table 8, EC_50_, EC_100_ and NOEC values for ABR test were obtained as 2.275, 4.430, and 0.839 mg/L, respectively. By comparing these tests, it can be concluded that, firstly, there is a meaningful relationship between them; secondly, toxicity of the effluent from the reactor and the toxicity of diazinon were confirmed by these new tests. In the study by Toolabi et al. (2017) reducing alamar blue (Resazurin) test of *Pseudomonas aerogenousa* bacteria was carried out in determining the toxicity of acetamiprid pesticide, In their study, it was found that alamar blue is not only a useful method for toxicity assessment, but it is also a very accurate and simple method.

In the current study, by assessing the toxicity tests on synthetic and real samples were determined, that Environmental factors such as temperature and turbidity is not affected on the performance of Alamar Blue test. In addition to the alamar blue test, the oxidation–reduction potential (ORP) to determine the activity of dehydrogenase enzyme *E. coli* was performed. Based on the results obtained in Table 8, the number and activity of *E. coli* bacteria was proportional to the amount of oxidation–reduction potential. According to the findings of this section, the alamar blue test was recognized as the most reliable, simplest, new method, rapid and economical for effluent toxicity assessment.

## Abbreviations

RT: retention time
ABR: alamar blue reduction
COD: chemical oxygen demand
ORP: oxidation reduction potential
NOEC: no observed effect concentration
EC: effective concentration
AOP: advanced oxidation process
GC-MS: gas chromatography Mass
HPLC: high performance liquid chromatography
